# Methods for Accurate Assessment of Myofiber Maturity During Skeletal Muscle Regeneration

**DOI:** 10.3389/fcell.2020.00267

**Published:** 2020-04-22

**Authors:** Yuki Yoshimoto, Madoka Ikemoto-Uezumi, Keisuke Hitachi, So-ichiro Fukada, Akiyoshi Uezumi

**Affiliations:** ^1^Muscle Aging and Regenerative Medicine, Tokyo Metropolitan Institute of Gerontology, Tokyo, Japan; ^2^Division for Therapies against Intractable Diseases, Institute for Comprehensive Medical Science, Fujita Health University, Toyoake, Japan; ^3^Project for Muscle Stem Cell Biology, Graduate School of Pharmaceutical Sciences, Osaka University, Suita, Japan

**Keywords:** skeletal muscle, muscle regeneration, muscle differentiation, satellite cells, muscle disease

## Abstract

Adult skeletal muscle has a remarkable ability to regenerate. Regeneration of mature muscle fibers is dependent on muscle stem cells called satellite cells. Although they are normally in a quiescent state, satellite cells are rapidly activated after injury, and subsequently proliferate and differentiate to make new muscle fibers. Myogenesis is a highly orchestrated biological process and has been extensively studied, and therefore many parameters that can precisely evaluate regenerating events have been established. However, in some cases, it is necessary to evaluate the completion of regeneration rather than ongoing regeneration. In this study, we establish methods for assessing the myofiber maturation during muscle regeneration. By carefully comparing expression patterns of several muscle regeneration-related genes, we found that expression of *Myozenin* (*Myoz1* and *Myoz3*), *Troponin I* (*Tnni2*), and *Dystrophin* (*Dmd*) is gradually increased as muscle regeneration proceeds. In contrast, commonly used regeneration markers such as *Myh3* and *Myh8* are transiently upregulated after muscle injury but their expression decreases as regeneration progresses. Intriguingly, upregulation of *Myoz1*, *Myoz3* and *Tnni2* cannot be achieved in cultured myotubes, indicating that these markers are excellent indicators to assess myofiber maturity. We also show that analyzing re-expression of Myoz1 and dystrophin in individual fiber during regeneration enables accurate assessment of myofiber maturity at the single-myofiber level. Together, our study provides valuable methods that are useful in evaluating muscle regeneration and the efficacy of therapeutic strategies for muscle diseases.

## Introduction

Skeletal muscle consists mainly of myofibers, which are large cylindrical cells with many nuclei. Myofibers are terminally differentiated post-mitotic cells; however, skeletal muscles possess a high ability to regenerate. Regeneration of mature myofibers is dependent on satellite cells. Satellite cells are mononucleated cells located between the plasma membrane of the myofiber and basal lamina. They normally remain in a quiescent state, but are activated upon muscle injury, and then they proliferate and differentiate to regenerate myofibers. Genetically engineered mice in which satellite cells are ablated show a complete lack of regenerative response (Lepper et al., 2011; Murphy et al., 2011; Sambasivan et al., 2011), indicating that satellite cells are absolutely required for muscle regeneration and cannot be compensated by other cell types. Furthermore, single-satellite cell transplantation revealed that these cells indeed possess self-renewal potential, in addition to the ability to differentiate into myofibers (Sacco et al., 2008). Thus, satellite cells are considered as definitive adult muscle stem cells.

Adult myogenesis is a highly ordered process in which satellite cells proliferate, differentiate, and generate new myofibers. Myogenic regulatory factors (MRFs) are important regulators of myogenesis and their expression is tightly regulated. Quiescent satellite cells do not express detectable levels of MyoD but they begin to express high levels of MyoD upon activation (Zammit et al., 2004). Expression of MyoD is maintained during the proliferation phase and continues until the early differentiation phase (Zammit et al., 2004). Myogenin is not expressed in quiescent satellite cells and proliferating undifferentiated myoblasts, but its expression is significantly upregulated when cells begin to differentiate (Bentzinger et al., 2012). Therefore, MyoD and Myogenin are commonly used as activation and differentiation markers of myogenesis, respectively. Expression levels of MRF4 are highest of the MRFs in adult mature muscle and are considered to reflect muscle fiber maturity (Bentzinger et al., 2012; Zammit, 2017). Adult muscle regeneration recapitulates many aspects of embryonic myogenesis, including expression of embryonic- or perinatal-type myosin heavy chain (MyHC) (Sartore et al., 1982). Thus, expression of these embryonic-type contractile proteins is a hallmark of muscle regeneration and is often used to detect activity of regeneration.

Expression of MRFs or embryonic or perinatal MyHC is useful to examine regenerating events. However, the most important goal in tissue regeneration is that the normal condition is restored. From this perspective, expression of the above described regeneration markers reflects conditions where muscle is still abnormal. In certain cases, therefore, assessment of “normality” becomes more important than evaluating regenerating events, especially in studies that examine the efficacy of therapeutic methods for degenerative muscle diseases. If diseased muscle is successfully treated and restored to its healthy state, expression of regeneration markers should be downregulated. Based on this notion, some studies examined downregulation of embryonic MyHC to assess therapeutic efficacy (Guiraud et al., 2019). However, little is known about indicators that directly reflect normality of muscle tissue.

Although experimental muscle regeneration is a highly ordered process, it is not completely synchronized, and thus there is a regional difference in the progression of regeneration within a single muscle. In a regeneration model of grafted muscle, it was reported that a radial gradient of regeneration is formed, with more mature muscle at the periphery and less mature muscle toward the center in the regenerating grafted muscle (Carlson and Gutmann, 1975). Likewise, other muscle regeneration models, including cardiotoxin injury models, do not show completely uniform regeneration, with some regions showing accelerated regeneration while other regions are in a delayed phase of regeneration. Therefore, it is important to develop a reliable method for evaluating muscle regeneration accurately and quantitatively, taking spatial non-uniformity of regeneration into account.

In this study, we carefully examined several regeneration-related markers during muscle regeneration. These analyses revealed that expression of *Myozenin* (*Myoz1* and *Myoz3*), *Troponin I* (*Tnni2*), and *Dystrophin* (*Dmd*) correlates very well with the progression of regeneration. Their expression highly reflects myofiber maturity because high expression of these genes can only be achieved in muscle tissue *in vivo* and not in cultured myotubes *in vitro*. We also developed a method that can distinguish advanced regenerating areas from delayed regenerating areas within single muscle, which enables accurate and quantitative evaluation of muscle regeneration. Our study provides useful information for the studies of muscle regeneration and therapy for muscle diseases.

## Materials and Methods

### Mice

C57BL/6 wild type mice were used to isolate satellite cells and to analyze muscle regeneration. DBA/2-mdx (D2-mdx) mice were provided from Central Institute for Experimental Animals in Japan. All animal experiments performed in this report were approved by the Animal Care and Use Committee of Tokyo Metropolitan Geriatric Hospital and Institute of Gerontology.

### Muscle Injury

Cardiotoxin (CTX, Sigma) was dissolved in sterile saline at a concentration of 10 μM. Tibialis anterior (TA) muscles of 2 to 3 month old mice were injected with 100 μl CTX. TA muscles were isolated at days 0, 3, 5, 7, and 14 of CTX injury, embedded in tragacanth gum, and frozen in liquid nitrogen-cooled isopentane.

### Satellite Cell Isolation

Isolation of mouse satellite cells was reported previously (Uezumi et al., 2016). Hind limb muscles were collected, minced and digested with 0.2% type II collagenase (Worthington) for 60 min at 37°C using a magnetic stirrer. Digested muscles were passed through an 18-gauge needle several times and further digested for 30 min at 37°C. Digested samples were filtered through a 100-μm cell strainer, and then through a 40-μm cell strainer. Cells were resuspended in washing buffer and labeled with APC-eFluor 780-conjugated rat anti-mouse CD45 (1:250) (Invitrogen), PE/Cy7-conjugated rat anti-mouse CD31 (1:250) (Biolegend), biotin-conjugated SM/C-2.6 (1:250) (Fukada et al., 2004), and PE-conjugated goat anti-mouse PDGFRα (15 μl/test) (R&D systems), followed by secondary staining with streptavidin-brilliant violet 421 (Biolegend) (1:250). CD31^–^CD45^–^PDGFRα^–^SM/C-2.6^+^ cells were sorted and collected as satellite cells with FACSAria II (BD Biosciences).

### Cell Culture

After cell sorting, satellite cells were seeded at a density of 1 × 10^4^ cells/well on a 48-well cell culture plate coated with Matrigel (BD Biosciences) in growth medium (GM) consisting of DMEM supplemented with 20% FBS, 1% penicillin-streptomycin, and 2.5 ng/μl bFGF (Katayama Chemical), and cultured at 37°C in 5% CO_2_ and 3% O_2_. After 4 days of culture in GM, GM was changed to differentiation medium (DM) consisting of DMEM with 5% horse serum. Then cells were maintained at 37°C in 5% CO_2_ and 20% O_2_ for 3 days to induce myogenic differentiation.

### RNA Extraction and Quantitative Reverse Transcription-PCR Analysis

Total RNA was extracted from cultured satellite cells and muscles using RNeasy Mini Kit (Qiagen) and miRNeasy Mini Kit (Qiagen), respectively. Pieces of muscle tissues collected from frozen TA muscles were crushed in QIAzol Lysis Reagent (Qiagen) using a Shakeman homogenizer (Bio Medical Science). Complementary DNA (cDNA) was synthesized using QuantiTect Transcription Kit (Qiagen). qRT-PCR was performed with SYBR Premix Ex Taq II (Takara) on a Takara Thermal Cycler Dice Real Time System (Takara) under the following cycling conditions: 94°C for 30 s followed by 40 cycles of amplification (94°C for 5 s, 60°C for 20 s, 72°C for 12 s) and dissociation curve analysis. For gene expression analysis in regenerating TA muscles and differentiating satellite cells, mRNA expression was normalized with *Cmas*. Relative mRNA expression was then calculated using the 2^–ΔΔ^ method. Specific primers used for qRT-PCR were listed in Supplementary Table 1. Primers for *Actb* were provided from QuantiTect Primer Assays Kit (Qiagen).

### Immunohistochemistry

Frozen transverse sections were cut at the thickness of 8 μm and fixed for 5 min in ice-cooled acetone. After blocking with M.O.M.^TM^ mouse IgG blocking reagent (Vector Laboratories), sections were incubated overnight at 4°C with primary antibodies diluted in M.O.M.^TM^ diluent. After washing with PBS, sections were stained with secondary antibodies. Primary and secondary antibodies used were listed in Supplementary Table 2. Nuclei were counterstained with DAPI (Dojindo), and stained muscles were mounted with SlowFade Diamond anti-fade reagent (Invitrogen). Fluorescent signals were detected with confocal laser scanning microscope systems TCS-SP8 (Leica). The same sections were stained with hematoxylin and eosin (HE) after capturing fluorescent images. HE images were taken with microscope AXIO (Carl Zeiss) equipped with a digital camera, Axiocam ERc 5s (Carl Zeiss).

### Quantitative Analysis of Mature Myofibers

Cross-sections were made by cutting at the mid-belly of TA muscle (at the position about 3 mm from proximal end of TA muscle). After immunostaining, fluorescent images of entire cross-sections were captured with fluorescent microscope system BZ-X710 (Keyence). Image recognition and quantification were performed by using the Hybrid Cell Count Application (Keyence). First, entire cross-sectional areas of TA muscle were measured. For quantification of Myoz1-positive area, Myoz1-stained area was recognized based on the intensity of Myoz1 staining by adjusting threshold. For quantification of dystrophin-positive area, dystrophin-stained sarcolemma was first recognized based on the intensity of dystrophin staining by adjusting threshold, and then dystrophin-positive fiber area was recognized by using inversion function. After recognition of Myoz1- and dystrophin-positive areas, the misrecognized small areas were excluded by adjusting lower limit in histogram function. Finally, errors in recognition step were corrected manually, and then Myoz1- and dystrophin-positive areas were measured. Myoz1- or dystrophin-positive area was divided by entire cross-sectional area to calculate percentage of area positive for each marker. Two side unpaired *t*-test was used to compare two groups.

### Statistical Analysis

Statistical significance was evaluated using GraphPad Prism 8.0 (GraphPad Software). One-way analysis of variance (ANOVA) followed by Tukey’s or Dunnett’s test was used to compare more than two groups.

## Results

### Optimum Internal Control Gene for Gene Expression Analysis During Muscle Regeneration

**FIGURE 1:**
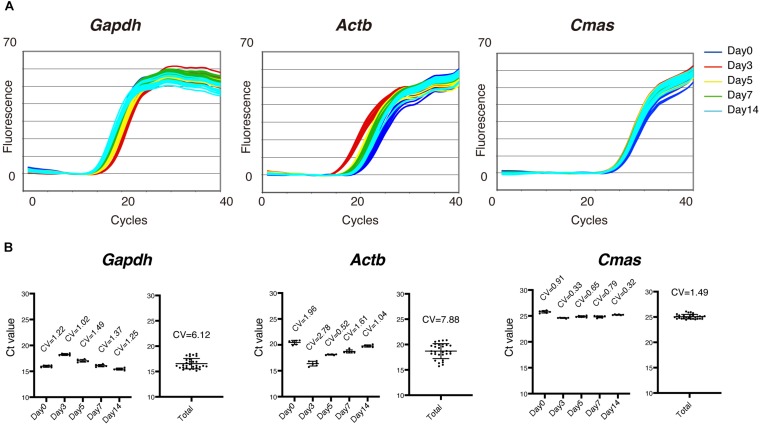
Optimum internal control genes for gene expression analysis during muscle regeneration. **(A)** Amplification curves of quantitative reverse transcription-PCR (qRT-PCR) for *Gapdh*, β*-actin* (*Actb*), and *Cmas* using total RNA extracted from intact and regenerating tibialis anterior (TA) muscles 3, 5, 7, and 14 days after CTX injury. **(B)** Cycle threshold (Ct) values of indicated time points and total data for *Gapdh*, *Actb*, and *Cmas* are shown as mean ± SD of *n* = 6 mice at each time point. Coefficient of variation (CV) is shown in the graphs. Note that Ct value of *Cmas* showed smaller CV than that of *Gapdh* or *Actb*.

We first analyzed the expression of several internal control genes by qRT-PCR to determine the optimum control genes for the most accurate gene expression analysis during muscle regeneration. As shown in Figure 1, *Gapdh* and *Actb* (also called β-actin), commonly used control genes, were highly variable in their expression during muscle regeneration (Figure 1). Therefore, these genes are not suitable as internal control genes to normalize expression of target genes. One pioneering study on comprehensive gene expression analysis during muscle regeneration had previously pointed out this problem and identified two genes that are stably expressed across all time points during muscle regeneration (Zhao and Hoffman, 2004). Those two genes are *Cmas* (also called as CMP-N-acetylneuraminic acid synthase) and *Eif3c* (called as NIPI-like protein). We thus examined the expression of *Cmas* and found relatively stable expression of this gene during muscle regeneration (Figure 1). Therefore, we decided to use *Cmas* as an internal control gene for gene expression analysis during muscle regeneration.

### Gradual Upregulation of *Myoz1*, *Myoz3*, *Tnni2*, and *Dmd* Reflects the Myofiber Maturity During Regeneration

**FIGURE 2:**
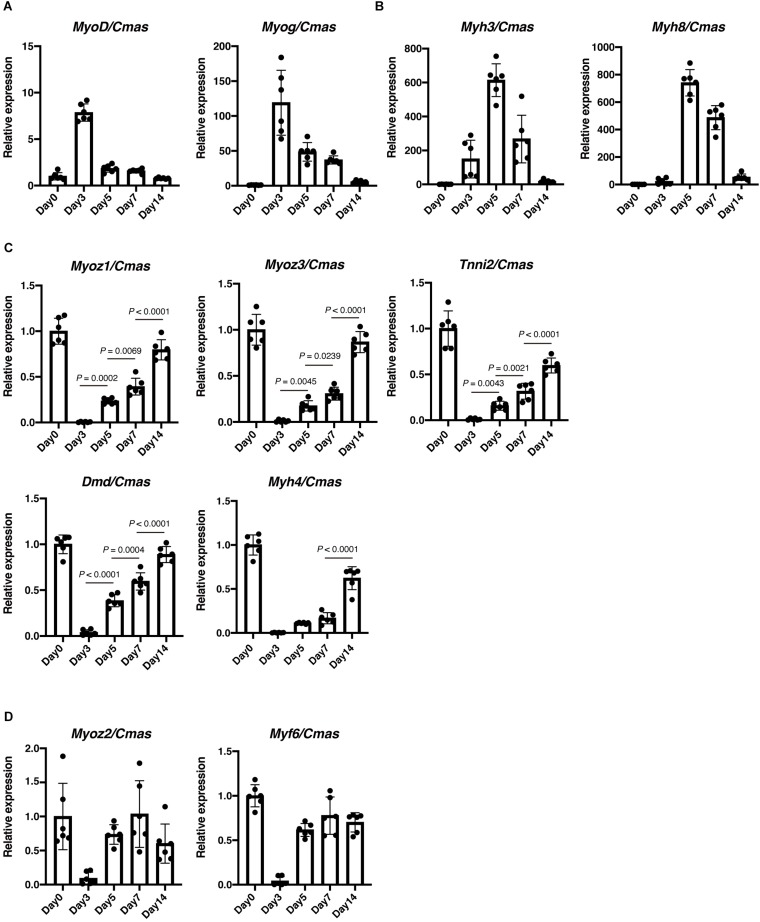
Expression of *Myoz1*, *Myoz3*, *Tnni2*, and *Dmd* correlates with the progression of muscle regeneration. Expression of *MyoD* and *Myog* **(A)**, *Myh3* and *Myh8* **(B)**, *Myoz1*, *Myoz3*, *Tnni2*, *Dmd* and *Myh4* **(C)**, *Myoz2* and *Myf6* **(D)** during muscle regeneration was examined by qRT-PCR. Data are shown as relative value to intact muscle (Day 0) and represent mean ± SD of *n* = 6 mice at each time point. Data on *Myoz1*, *Myoz3*, *Tnni2*, *Dmd*, and *Myh4* from day 3 to day 14 were analyzed by ANOVA followed by Tukey’s test to evaluate statistical difference.

We next examined expression of several regeneration-related genes. As expected, expression of *MyoD* and *Myogenin* were highly induced upon muscle injury, and gradually downregulated thereafter (Figure 2A). We also observed similar dynamics in the expression of embryonic-type contractile genes. As shown in Figure 2B, expression of *Myh3* and *Myh8* was detected at day 3 of muscle injury, reached its peak at day 5, and then decreased to levels comparable to intact muscle. Thus, expression of above-described genes is transient during muscle regeneration and therefore does not reflect completion of regeneration accurately. Zhao et al. (2002) performed temporal gene expression profiling of muscle regeneration and showed that expression of muscle structural component genes is downregulated at early stages and then upregulated at late stages of muscle regeneration. Those include *Myozenin*, which encodes a Z-disk associated protein myozenin, and *Tnni2*, which encodes a fast skeletal type troponin I, a protein responsible for the calcium-dependent regulation of muscle contraction. Therefore, we examined expression of these muscle structural component genes. Expression of *Myoz1*, *Myoz3*, and *Tnni2* was sharply downregulated at day 3 of muscle injury, and then gradually upregulated as regeneration proceeded (Figure 2C), indicating that expression of these genes well reflects the extent of muscle regeneration. We also analyzed expression of *Dmd*, which encodes a dystrophin protein, and *Myh4*, which encodes a MyHC-IIb, a predominant type of MyHC expressed in TA muscle (Kammoun et al., 2014). Similar to *Myoz1*, *Myoz3* and *Tnni2*, expression of *Dmd* correlated well with the progression of regeneration (Figure 2C). Although *Myh4* showed a similar expression pattern, it reflected the extent of regeneration less accurately because there was no statistically significant difference in its expression levels between day 3 and day5, or day 5 and day 7 (Figure 2C). In contrast to these genes, expression of *Myoz2* and *Myf6* did not reflect muscle maturity (Figure 2D). These results clearly show that *Myoz1*, *Myoz3*, *Tnni2*, and *Dmd* are excellent markers for the assessment of myofiber maturity during muscle regeneration.

### High Level Expression of *Myoz1*, *Myoz3*, and *Tnni2* Cannot Be Achieved in Cultured Myotubes

**FIGURE 3:**
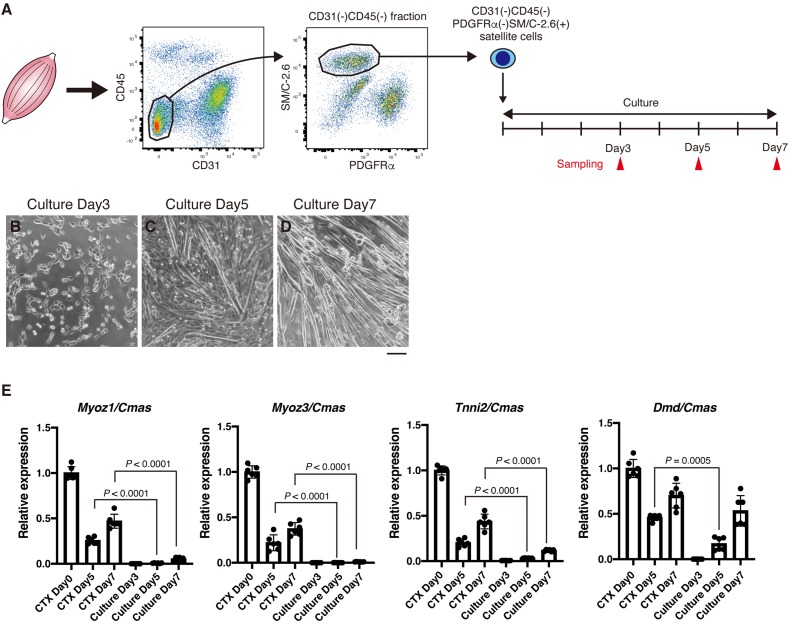
*In vitro* cultured myotubes do not express high levels of *Myoz1*, *Myoz3*, and *Tnni2*. **(A)** Scheme of satellite cell isolation and culture. **(B–D)** Isolated satellite cells were cultured in GM for 4 days, then induced to differentiate into myotubes in DM. Representative images of cultured cells were taken at indicated time points. **(E)** Expression of *Myoz1*, *3*, *Tnni2*, and *Dmd* in intact (CTX Day 0), regenerating TA muscle (CTX Days 5 and 7) and cultured satellite cells was examined by qRT-PCR. Data are shown as relative value to intact TA muscle and represent the mean ± SD from independent experiments (*n* = 6). Data from regenerating TA muscle (CTX Days 5 and 7) and cultured cells were analyzed by ANOVA followed by Tukey’s test to evaluate statistical difference. Scale bar: 100 μm **(B–D)**.

Results described above strongly suggest that expression of *Myoz1*, *Myoz3*, *Tnni2*, and *Dmd* correlates with myofiber maturity. It is well-known that cultured myotubes cannot mature into myofibers. To further confirm the relationship between expression of *Myoz1*, *Myoz3*, *Tnni2*, and *Dmd* and myofiber maturity, we examined the expression of these genes during myogenesis of cultured satellite cells. Satellite cells were FACS-sorted from hind limb muscles and cultured *in vitro* to obtain myotubes (Figure 3A). During the first 4 days of the growth period, satellite cells became activated, and they proliferated extensively (Figure 3B). Upon induction of differentiation, they rapidly formed myotubes at day 5 of culture, and generated numerous myotubes by day 7 as they further differentiated (Figures 3C,D). In CTX muscle regeneration model, satellite cells proliferate extensively within 2 to 3 days of injury and begin to form regenerated myofibers approximately 5 days after injury, and regenerated myofibers mature afterward (Hawke and Garry, 2001). Thus, proliferation period and timing of differentiation of satellite cells are similar between *in vitro* myogenesis and *in vivo* regeneration model. As expected, undifferentiated myoblasts expressed very low levels of *Myoz1*, *Myoz3*, *Tnni2*, and *Dmd* similarly to CTX-injected muscle at day 3 (Figures 2C, 3E). In later time points, expression levels of *Myoz1*, *Myoz3* and *Tnni2* remained quite low compared to levels in intact and regenerating (CTX day 5 and day 7) TA muscles *in vivo* (Figure 3E). Although expression of *Dmd* remained low levels until day 5 of culture, its expression in myotubes increased to the levels comparable to *in vivo* regenerating muscle at day 7 (Figure 3E). These data further reinforce the view that expression of *Myoz1*, *Myoz3*, and *Tnni2* well-reflects myofiber maturity that cannot be achieved in cultured myotubes.

### Centrally Nucleated Fibers With Myoz1 and Dystrophin Expression Identify Areas of Advanced Regeneration

**FIGURE 4:**
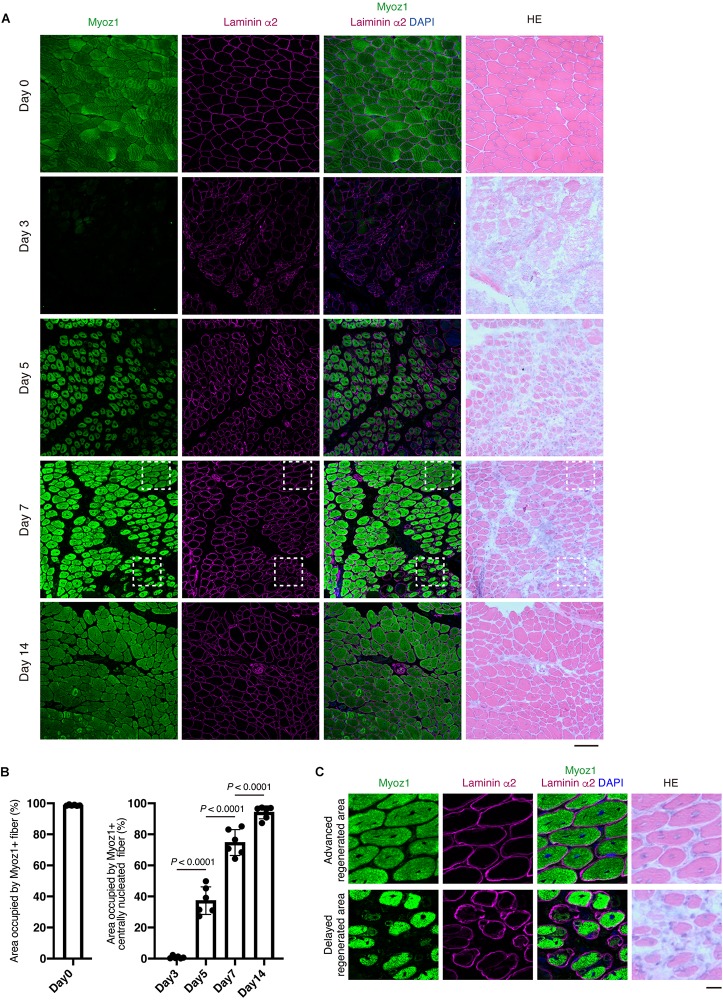
Re-expression of Myoz1 protein is closely associated with the extent of myofiber regeneration. **(A)** TA muscle sections from the indicated time points were subjected to immunofluorescent staining for Myoz1 (green) and Laminin α2 (magenta) followed by HE staining. **(B)** Area occupied by Myoz1+ fibers in intact muscle (Day 0) and Myoz1+ centrally nucleated fibers in regenerating muscle was quantified. Data represent the mean ± SD of *n* = 6 mice at each time point. Data from regenerating muscle (Day 3 to Day 14) were analyzed by ANOVA followed by Tukey’s test to evaluate statistical difference. **(C)** Magnified images of boxed areas in **(A)**. Upper panels show area with advanced regeneration and lower panels show area with delayed regeneration. Scale bars: 100 μm **(A)** and 20 μm **(C)**.

**FIGURE 5:**
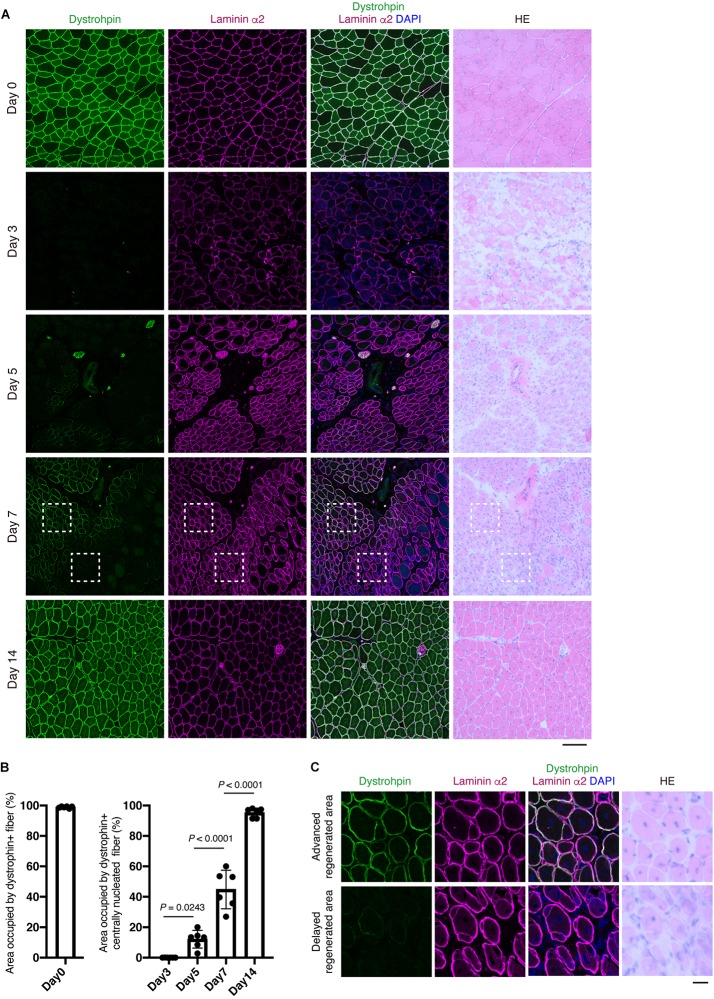
Re-expression of dystrophin protein at the plasma membrane is closely associated the extent of myofiber regeneration. **(A)** TA muscle sections from the indicated time points were subjected to immunofluorescent staining for Dystrophin (green) and Laminin α2 (magenta) followed by HE staining. **(B)** Area occupied by Dystrophin+ fibers in intact muscle (Day 0) and Dystrophin+ centrally nucleated fibers in regenerating muscle was quantified. Data represent the mean ± SD of *n* = 6 mice at each time point. Data from regenerating muscle (Day 3 to Day 14) were analyzed by ANOVA followed by Tukey’s test to evaluate statistical difference. **(C)** Magnified images of boxed areas in **(A)**. Upper panels show area with advanced regeneration and lower panels show area with delayed regeneration. Scale bars: 100 μm **(A)** and 20 μm **(C)**.

Evaluating *Myoz1*, *Myoz3*, *Tnni2*, and *Dmd* expression would be very useful for assessing the degree of muscle regeneration at the whole-tissue level. However, in some cases, it is necessary to assess muscle regeneration at the single-myofiber level because muscle regeneration is not a uniform process, with some regions showing advanced regeneration while other regions showing a delayed phase of regeneration. To overcome this problem, we developed a method that can accurately assess this spatial non-uniformity of regeneration. Centrally located nuclei are commonly used as an index of regenerated myofibers. However, central nuclei already exist in nascent myotubes, precluding its use as a reference index when assessing myofiber maturity. Among the markers whose expression correlate well with the progression of regeneration (*Myoz1*, *Myoz3*, *Tnni2*, and *Dmd*), we could obtain clear staining results for Myoz1 and dystrophin proteins. Because Myoz1 is a Z-disk associated protein, Myoz1-stained muscle showed sarcomere pattern, suggesting the specificity of the antibody used in this study (Supplementary Figure 1). We found that Myoz1 expression disappears upon muscle injury but gradually reappears as muscle regeneration proceeds (Figures 4A,B). Intriguingly, although well-differentiated large centrally nucleated fibers were strongly positive for Myoz1, small basophilic nascent myotubes were scantly positive or negative for Myoz1 even in contiguous areas of the same muscle (Figure 4C). Dystrophin staining resulted in similar expression pattern, although dystrophin re-expression tended to be restricted to more mature myofibers (Figure 5). These results indicate that centrally nucleated myofiber with recovered Myoz1 or dystrophin expression is useful for assessing the spatial non-uniformity of muscle regeneration at single-myofiber level.

### Re-expression of Myoz1 and Dystrophin Is a Good Indicator of Myofiber Maturity at the Single-Myofiber Level

**FIGURE 6:**
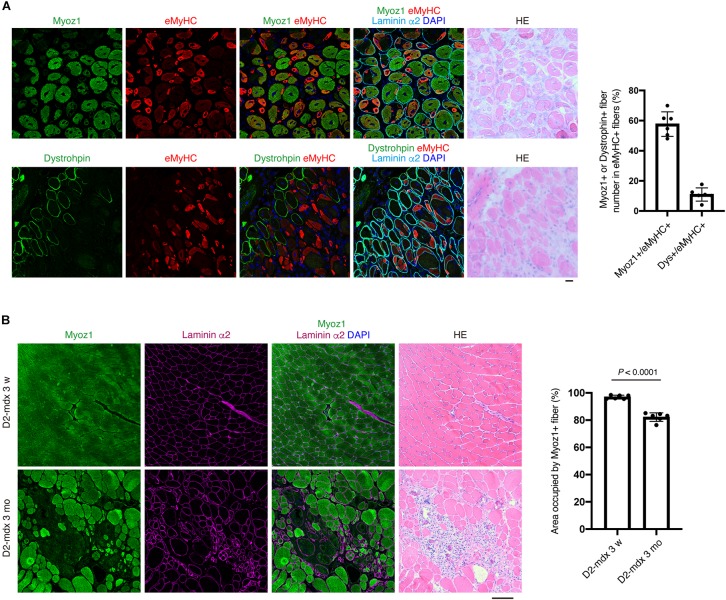
Expression of Myoz1 and dystrophin protein is a good indicator of myofiber maturity at the single-fiber level. **(A)** TA muscle section from day 7 of CTX injection was subjected to immunofluorescent staining for Myoz1 or Dystrophin (green), eMyHC (red) and Laminin α2 (cyan) followed by HE staining. Graph shows percentage of Myoz1+ or Dystrophin+ fiber number in eMyHC+ fibers. Data represent the mean ± SD of *n* = 6 mice. **(B)** Quadriceps muscle section from D2-mdx mice at 3 weeks or 3 months of age was subjected to immunofluorescent staining for Myoz1 (green) and Laminin α2 (magenta) followed by HE staining. Area occupied by Myoz1+ fibers was quantified. Data represent the mean ± SD of *n* = 6 mice at each time point. Data was analyzed by two-sided unpaired *t*-test to evaluate statistical difference. Scale bars: 20 μm **(A)** and 100 μm **(B)**.

To further understand the relationship between Myoz1 or dystrophin expression and myofiber maturity, we examined embryonic MyHC (eMyHC) expression, which is transiently upregulated in immature myofibers but downregulated as myofibers mature (d’Albis et al., 1988). Approximately 40 and 90% of eMyHC-positive immature myofibers were negative for Myoz1 and dystrophin, respectively (Figure 6A), indicating that re-expression of these markers occurs in myofibers with advanced maturation stage.

We also examined usefulness of these markers in evaluating disease progression of D2-mdx mice, a severe mouse model of Duchenne muscular dystrophy (DMD) (Fukada et al., 2010). Muscle of mdx background appears normal until approximately 3–4 weeks of age, but myofibers undergo massive degeneration afterward (DiMario et al., 1991). Because D2-mdx mice lack dystrophin expression, we examined expression of Myoz1 before and after disease onset. At 3 weeks of age, all myofibers of D2-mdx mice appeared normal and uniformly expressed Myoz1 (Figure 6B). After onset of symptoms, however, small immature myofibers located in degenerated area were scantly positive or negative for Myoz1, and total Myoz1-positive area was significantly decreased in ratio compared with pre-symptomatic stage (Figure 6B). These results indicate that expression of Myoz1 and dystrophin is a good indicator of myofiber maturity and useful for evaluating “normality” of myofiber in pathological conditions.

## Discussion

In this study, we describe methods for the assessment of myofiber maturity during skeletal muscle regeneration. Expression of *Myoz1*, *Myoz3*, *Tnni2*, and *Dmd* significantly correlates with progression of muscle regeneration, and therefore, these genes are quite useful to quantify and evaluate the extent of muscle regeneration at the whole-muscle tissue level. Meanwhile, re-expression of Myoz1 and dystrophin is an excellent indicator for the assessment of myofiber maturity at the single-fiber level.

Myozenin is specifically expressed in striated muscle and localized at Z-disks (Faulkner et al., 2000; Takada et al., 2001). Myozenin is reported to interact with other Z-disk proteins including a-actinin, filamin-C, telethonin and myotilin, and thought to be involved in the connection between the contractile apparatus and the sarcolemma (Faulkner et al., 2000; Frey et al., 2000; Takada et al., 2001; Frey and Olson, 2002; Gontier et al., 2005). In addition to its role as a structural protein, Myoz1 was shown to modulate calcineurin/NFAT activity (Frey et al., 2008), raising the possibility that Myozenin functions as a signaling molecule. In this study, we showed that expression of *Myoz1* and *Myoz3* is gradually upregulated as myofibers mature during muscle regeneration, and cultured myotubes do not express these genes at high levels. Therefore, it would be reasonable to assume that Myozenin plays some functional role in myofiber maturation.

We demonstrated that dystrophin begins to be re-expressed when myofibers mature in the late phase of muscle regeneration. Therefore, dystrophin re-expression closely reflects myofiber maturity. Although this method will provide a very powerful means of assessing muscle regeneration, there is a certain limitation. Because this evaluation method depends on dystrophin expression, it cannot be used in dystrophin-deficient conditions such as DMD. Evaluating Myoz1 expression offers an alternative method in such a situation as we showed in this study. However, definitive therapy for DMD is restoration of dystrophin expression. Therefore, dystrophin expression is commonly studied in research when evaluating the effects of DMD therapy. If the therapeutic strategy is based on endogenous gene expression machinery (such as exon skipping or gene editing therapy) and not on forced expression, restored dystrophin expression reflects not only proof of principle but also maturity of treated myofibers. Thus, our study provides additional rationale for examining dystrophin expression in DMD therapy research.

Methods described here are based on gene expression and histological analyses, but one of the most important functions of skeletal muscle is to contract for force generation. Therefore, measurement of contractile ability is one of the best ways to evaluate skeletal muscle property. However, evaluating contractile properties is accompanied by some technical difficulties. Our methods presented here are relatively stable and easy to perform. In addition, markers used in our methods seem to be functionally important because myozenin and troponin I are associated with contractile apparatus, and dystrophin is a causative gene for DMD. Thus, we believe that our methods provide convenient opportunity to assess myofiber maturity.

In conclusion, our study provides meaningful information that can be applied for accurate and quantitative assessment of muscle regeneration and effectiveness of therapy for muscle diseases.

## Data Availability Statement

All datasets generated for this study are included in the article/Supplementary Material.

## Ethics Statement

The animal study was reviewed and approved by The Animal Care and Use Committee of Tokyo Metropolitan Geriatric Hospital and Institute of Gerontology.

## Author Contributions

YY performed qPCR, immunostaining and cell culture experiments, and analyzed the data. MI-U and KH induced muscle regeneration and retrieved samples. MI-U performed FACS experiments. AU and SF interpreted results and coordinated the project. AU conceived the whole project and wrote the manuscript.

## Conflict of Interest

The authors declare that the research was conducted in the absence of any commercial or financial relationships that could be construed as a potential conflict of interest.

The reviewer TS declared a shared affiliation, with no collaboration, with the author KH, to the handling Editor, at the time of review.

## References

[B1] Bentzinger C. F. Wang Y. X. Rudnicki M. A. (2012). Building muscle: molecular regulation of myogenesis. *Cold Spring Harb. Perspect. Biol.* 4:a008342. 10.1101/cshperspect.a008342 22300977 PMC3281568

[B2] Carlson B. M. Gutmann E. (1975). Regneration in free grafts of normal and denervated muscles in the rat: morphology and histochemistry. *Anat. Rec.* 183 47–62. 10.1002/ar.1091830106 126650

[B3] d’Albis A. Couteaux R. Janmot C. Roulet A. Mira J. C. (1988). Regeneration after cardiotoxin injury of innervated and denervated slow and fast muscles of mammals. myosin isoform analysis. *Eur. J. Biochem.* 174 103–110. 10.1111/j.1432-1033.1988.tb14068.x 3371354

[B4] DiMario J. X. Uzman A. Strohman R. C. (1991). Fiber regeneration is not persistent in dystrophic (MDX) mouse skeletal muscle. *Dev. Biol.* 148 314–321. 10.1016/0012-1606(91)90340-9 1936568

[B5] Faulkner G. Pallavicini A. Comelli A. Salamon M. Bortoletto G. Ievolella C. (2000). FATZ, a filamin-, actinin-, and telethonin-binding protein of the Z-disc of skeletal muscle. *J. Biol. Chem.* 275 41234–41242. 10.1074/jbc.M007493200 10984498

[B6] Frey N. Frank D. Lippl S. Kuhn C. Kogler H. Barrientos T. (2008). Calsarcin-2 deficiency increases exercise capacity in mice through calcineurin/NFAT activation. *J. Clin. Invest.* 118 3598–3608. 10.1172/jci36277 18846255 PMC2564612

[B7] Frey N. Olson E. N. (2002). Calsarcin-3, a novel skeletal muscle-specific member of the calsarcin family, interacts with multiple Z-disc proteins. *J. Biol. Chem.* 277 13998–14004. 10.1074/jbc.M200712200 11842093

[B8] Frey N. Richardson J. A. Olson E. N. (2000). Calsarcins, a novel family of sarcomeric calcineurin-binding proteins. *Proc. Natl. Acad. Sci. U.S.A.* 97 14632–14637. 10.1073/pnas.260501097 11114196 PMC18970

[B9] Fukada S. Higuchi S. Segawa M. Koda K. Yamamoto Y. Tsujikawa K. (2004). Purification and cell-surface marker characterization of quiescent satellite cells from murine skeletal muscle by a novel monoclonal antibody. *Exp. Cell Res.* 296 245–255. 10.1016/j.yexcr.2004.02.018 15149854

[B10] Fukada S. Morikawa D. Yamamoto Y. Yoshida T. Sumie N. Yamaguchi M. (2010). Genetic background affects properties of satellite cells and mdx phenotypes. *Am. J. Pathol.* 176 2414–2424. 10.2353/ajpath.2010.090887 20304955 PMC2861106

[B11] Gontier Y. Taivainen A. Fontao L. Sonnenberg A. van der Flier A. Carpen O. (2005). The Z-disc proteins myotilin and FATZ-1 interact with each other and are connected to the sarcolemma via muscle-specific filamins. *J. Cell Sci.* 118(Pt 16) 3739–3749. 10.1242/jcs.02484 16076904

[B12] Guiraud S. Edwards B. Squire S. E. Moir L. Berg A. Babbs A. (2019). Embryonic myosin is a regeneration marker to monitor utrophin-based therapies for DMD. *Hum. Mol. Genet.* 28 307–319. 10.1093/hmg/ddy353 30304405 PMC6322073

[B13] Hawke T. J. Garry D. J. (2001). Myogenic satellite cells: physiology to molecular biology. *J. Appl. Physiol. (1985)* 91 534–551. 10.1152/jappl.2001.91.2.534 11457764

[B14] Kammoun M. Cassar-Malek I. Meunier B. Picard B. (2014). A simplified immunohistochemical classification of skeletal muscle fibres in mouse. *Eur. J. Histochem.* 58:2254. 10.4081/ejh.2014.2254 24998919 PMC4083319

[B15] Lepper C. Partridge T. A. Fan C. M. (2011). An absolute requirement for Pax7-positive satellite cells in acute injury-induced skeletal muscle regeneration. *Development* 138 3639–3646. 10.1242/dev.067595 21828092 PMC3152922

[B16] Murphy M. M. Lawson J. A. Mathew S. J. Hutcheson D. A. Kardon G. (2011). Satellite cells, connective tissue fibroblasts and their interactions are crucial for muscle regeneration. *Development* 138 3625–3637. 10.1242/dev.064162 21828091 PMC3152921

[B17] Sacco A. Doyonnas R. Kraft P. Vitorovic S. Blau H. M. (2008). Self-renewal and expansion of single transplanted muscle stem cells. *Nature* 456 502–506. 10.1038/nature07384 18806774 PMC2919355

[B18] Sambasivan R. Yao R. Kissenpfennig A. Van Wittenberghe L. Paldi A. Gayraud-Morel B. (2011). Pax7-expressing satellite cells are indispensable for adult skeletal muscle regeneration. *Development* 138 3647–3656. 10.1242/dev.067587 21828093

[B19] Sartore S. Gorza L. Schiaffino S. (1982). Fetal myosin heavy chains in regenerating muscle. *Nature* 298 294–296. 10.1038/298294a0 7045696

[B20] Takada F. Vander Woude D. L. Tong H. Q. Thompson T. G. Watkins S. C. Kunkel L. M. (2001). Myozenin: an alpha-actinin- and gamma-filamin-binding protein of skeletal muscle Z lines. *Proc. Natl. Acad. Sci. U.S.A.* 98 1595–1600. 10.1073/pnas.041609698 11171996 PMC29302

[B21] Uezumi A. Kasai T. Tsuchida K. (2016). Identification, isolation, and characterization of mesenchymal progenitors in Mouse and human skeletal muscle. *Methods Mol. Biol.* 1460 241–253. 10.1007/978-1-4939-3810-0_17 27492177

[B22] Zammit P. S. (2017). Function of the myogenic regulatory factors Myf5, MyoD, Myogenin and MRF4 in skeletal muscle, satellite cells and regenerative myogenesis. *Semin. Cell Dev. Biol.* 72 19–32. 10.1016/j.semcdb.2017.11.011 29127046

[B23] Zammit P. S. Golding J. P. Nagata Y. Hudon V. Partridge T. A. Beauchamp J. R. (2004). Muscle satellite cells adopt divergent fates: a mechanism for self-renewal? *J. Cell Biol.* 166 347–357. 10.1083/jcb.200312007 15277541 PMC2172269

[B24] Zhao P. Hoffman E. P. (2004). Embryonic myogenesis pathways in muscle regeneration. *Dev. Dyn.* 229 380–392. 10.1002/dvdy.10457 14745964

[B25] Zhao P. Iezzi S. Carver E. Dressman D. Gridley T. Sartorelli V. (2002). Slug is a novel downstream target of MyoD. Temporal profiling in muscle regeneration. *J. Biol. Chem.* 277 30091–30101. 10.1074/jbc.M202668200 12023284

