# Supplementary material for: Methods for Accurate Assessment of Myofiber Maturity During Skeletal Muscle Regeneration
Source: Front Cell Dev Biol. 2020 Apr 22;8:267. doi: 10.3389/fcell.2020.00267 (PMC7188918; doi:10.3389/fcell.2020.00267)
Supplement: Supplementary file 1 [file Data_Sheet_1.pdf]

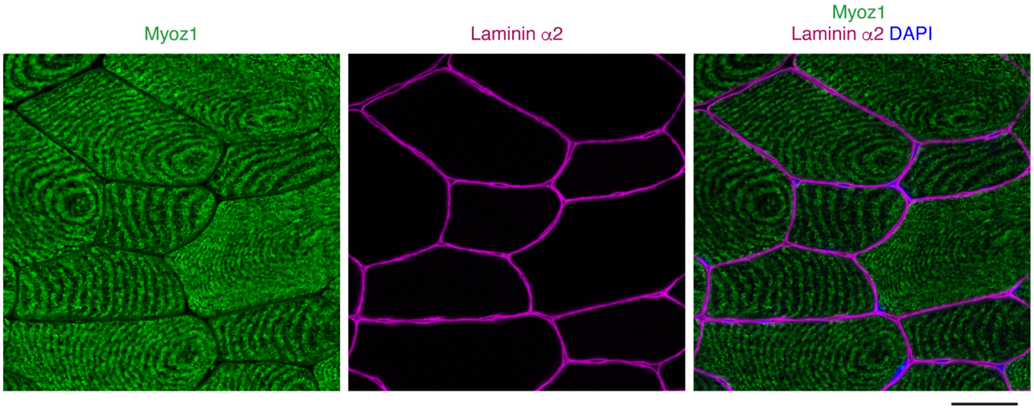

Supplementary Figure 1

Supplementary Figure 1. Myoz1 staining reveals sarcomere pattern in myofiber. TA muscle section was subjected to immunofluorescent staining for Myoz1 (green) and Laminin a2 (magenta). Scale bar, 25 μm

Supplementary Table 1. Primers used in qRT-PCR

Gene	Direction	Sequence
Myoz1	Forward	5'-GGAACTTGGCATTGACCTACTG-3'
	Reverse	5'-AAACTTGGGCATCTGGAAGG-3'
Myoz2	Forward	5'-CAGAACTGCGGGATTACAGG-3'
	Reverse	5'-GTCTGCCCGAAAGAGGATTG-3'
Myoz3	Forward	5'-TGGCAGCAGAAGTCACACTC-3'
	Reverse	5'-AGTTCCAAGCCACTGAAGGAC-3'
Tnni2	Forward	5'-CGGAGACAGCACCTGAAGAG-3'
	Reverse	5'-AGACATGGAGCCTGGGATG-3'
Dmd	Forward	5'-GGAAGAAGTAGAGGACTGTTATG-3'
	Reverse	5'-AGGTCTAGGAGGCGTTTTCC-3'
Myh4	Forward	5'-AAACCACCTCAGAGTTGTGGA-3'
	Reverse	5'-GTTCCGAAGGTTCCTGATTGC-3'
Myf6	Forward	5'-ATCAGCTACATTGAGCGTCTACA-3'
	Reverse	5'-CCTGGAATGATCCGAAACACTTG-3'
MyoD	Forward	5'-CCACTCCGGGACATAGACTTG-3'
	Reverse	5'-AAAAGCGCAGGTCTGGTGAG-3'
Myog	Forward	5'-CAGTACATTGAGCGCCTACAG-3'
	Reverse	5'-GGACCGAACTCCAGTGCAT-3'
Myh3	Forward	5'-ACACGGATCAGAGAGCTGGA-3'
	Reverse	5'-CCTTAACACGCCGCTCATAC-3'
Myh8	Forward	5'-CATCCACGCAGCAGATTGA-3'
	Reverse	5'-CGCAGCAGATCACAGTCGT-3'
Cmas	Forward	5'-CAAAGGCATCCCACTGAAGA-3'
	Reverse	5'-CCCACACACTCTGGAAGACC-3'
Gapdh	Forward	5'-CCTGGAGAAACCTGCCAAGTATG-3'
	Reverse	5'-AGAGTGGGAGTTGCTGTTGAAGTC-3'

Supplementary Table 2. Antibodies used in this study

Antibody	Dilution	Supplier
Rabbit polyclonal anti-Dystrophin	1:800	Abcam
Mouse monoclonal anti-embryonic Myosin heavy chain	1:2	Clone: F1.652, DSHB
Rabbit polyclonal anti-Myoz1	1:250	Sigma
Rat monoclonal anti-Laminin α2	1:200	Clone: 4H8-2, SantaCruz Biotechnology
Alexa Fluor 488-conjugated donkey anti-rabbit IgG	1:1000	Jackson ImmunoResarch
Cy3-conjugated donkey anti-mouse IgG	1:1000	Jackson ImmunoResarch
Alexa Fluor 647-conjugated chick anti-rat IgG	1:1000	Molecular Probes
